# Multimorbidity and care dependence in older adults: a longitudinal analysis of findings from the 10/66 study

**DOI:** 10.1186/s12889-019-6961-4

**Published:** 2019-05-16

**Authors:** Jianan Bao, Kia-Chong Chua, Matthew Prina, Martin Prince

**Affiliations:** Health Service and Population Research, Institute of Psychiatry, Psychology & Neuroscience, King’s College London, London, UK

**Keywords:** Multimorbidity, Care dependence, Longitudinal, Older adults, China, Latin America, Chronic diseases, Depression, Dementia, Anxiety

## Abstract

**Background:**

In an ageing world facing an epidemic of chronic diseases, there is great interest in the burden of multimorbidity on individuals and caregivers, yet no studies have examined the longitudinal association between multimorbidity and care dependence in low and middle income countries. Mental and cognitive disorders are associated with dependence but little is known about their role in the pathway to dependence in the context of multimorbidity. This study aims to determine (1) the association of multimorbidity with the onset of care dependence in older adults, accounting for mortality and controlling for sociodemographic factors, and (2) the independent effects of physical multimorbidity, mental and cognitive disorders.

**Methods:**

A population-based cohort study of people aged 65 years and older in six countries in Latin America, and China. Data on chronic conditions and sociodemographic factors were collected at baseline. Multimorbidity was ascertained as a count of up to 15 mental, cognitive and physical health conditions. Dependence was ascertained through informant interviews at baseline and follow-up. We used competing risk regression to assess the association between multimorbidity and the onset of care dependence, acknowledging the possibility of dependence-free death. We also assessed the independent effects of physical multimorbidity and depression, anxiety and dementia individually.

**Results:**

12,965 participants, with no needs for care at baseline, were followed up for a median of 3.0–4.9 years. Each unit increase in multimorbidity count increased the cumulative risk of dependence by 20% in the fully adjusted model. Age was the only variable to confound this relationship. Physical multimorbidity was associated with only a modest increased risk of care dependence. Dementia, depression and anxiety were independently associated with incident care dependence at every level of physical multimorbidity, and depression and anxiety attenuated the effect of physical multimorbidity.

**Conclusion:**

Multimorbidity consistently predicts care dependence with little variation between countries. Physical multimorbidity imparts a lower risk than multimorbidity with mental and cognitive disorders included. Mental and cognitive disorders independently increase the risk of care dependence. Comprehensive and holistic assessment of disorders of body, brain and mind can help to identify older people at high risk of care dependence.

## Background

More than half of older people around the world have more than one chronic condition [1, 2]. This accumulation of manifest diseases is otherwise known as multimorbidity. As populations age, and the burden from noncommunicable diseases increases, there is a growing focus on the goal of postponing the onset of morbidity due to chronic conditions [3]. This is especially relevant for low and middle income countries (LMICs) undergoing rapid demographic transition, alongside changes to traditional networks providing support and care for older persons [4].

The burden of multimorbidity can often feel like more than the sum of its parts, the physical and emotional demands of which are often shared with caregivers [5]. The requirement of additional help from other people is referred to as care dependence. Whilst the caregiving can be a positive experience, it can also be a source of psychological strain and financial burden [6]. In LMIC families with care dependent older people, caregivers often need to give up work or education, leading to significant financial vulnerability compounded by an increased risk of catastrophic healthcare expenditure [7]. Caregivers face unique challenges when caring for people with multimorbidity: they experience less support from healthcare services compared to caring for those with single illnesses [8], and take on the additional work that comes with dealing with the complexity, e.g. managing polypharmacy and adherence [9]. Care dependence is therefore a useful outcome for policy and practice in the context of multimorbidity [10, 11].

The risk of disability in those with multimorbidity has been explored [1, 12, 13] and the risk of dependence in relation to frailty has been investigated [14, 15] but there have been no longtidudinal studies from LMICs looking at the risk of care dependence in people with multimorbidity, and only one study from a high income country (HIC) examining this relationship [16]. Aside from a marked association with older age [17], care dependence has important social determinants, including education and socioeconomic position [6, 10]. It is therefore relevant, while controlling for these factors, to compare associations of multimorbidity with incident dependence among countries with diverse economic and health systems.

Whilst it is known that mental and cognitive disorders contribute significantly to dependence [6, 16, 18], that they often cluster together [19], and are more prevalent in people with multimorbidity [20], their role in the pathway to dependence, relative to that of physical multimorbidity, has not been explored. It is possible that they may confound or mediate any effect of physical multimorbidity on incident dependence. There remain knowledge gaps about the way in which different diseases relate to each other, reflected in the vertical nature of most clinical guidelines that only consider evidence applying to single diseases [21].

This study therefore seeks to investigate:

(1) the risk of care dependence in older adults with multimorbidity, accounting for mortality and controlling for sociodemographic factors, and (2) the independent effects of physical multimorbidity and individual mental and cognitive disorders.

## Methods

### Participants and procedures

Baseline data was collected between 2003 and 2007, and follow-up data between 2008 and 2010 in urban catchment area sites in Cuba (Havana and Matanzas), Dominican Republic (Santo Domingo), Puerto Rico (Bayamon), and Venezuela (Caracas), and urban and rural sites in Peru (Lima and Canete), Mexico (Mexico City and Morelos) and China (Beijing and Daxing). All those normally resident in the catchment areas, and aged 65 years or older were eligible to participate. Data collection included interviews with participants and key informants, and a physical examination, administered at baseline and follow-up. Further details can be found in the published cohort profile [22].

### Measures

Assessment of multimorbidity included the ascertainment of 15 chronic conditions. The individual conditions were assessed as self-reported impairments (vision, hearing, joints, skin, gastrointestinal), report of characteristic symptoms (angina, stroke, chronic obstructive pulmonary disease), self-reported diagnoses (stroke, hypertension, diabetes, ischaemic heart disease, heart failure, heart valve disease) and/ or following clinical assessment (depression, anxiety, dementia, hypertension) as follows:

- Stroke – self-report, confirmed by the interviewer as having had characteristic symptoms persisting for more than 24 h.
- Hypertension – self-report of diagnosed hypertension, and/ or meeting World Health Organization/ International Society of Hypertension (WHO-ISH) criteria based on recorded blood pressure level.
- Diabetes – self-report of diagnosed diabetes, or taking insulin or hypoglycaemic medication.
- Ischaemic heart disease – self-report of heart attack, angina, or “heart trouble” or angina that interferes with activities a little or a lot.
- Heart failure – self-report.
- Valve disease – self-report.
- Chronic obstructive pulmonary disease (COPD) – clinical assessment based on following self-reported symptoms: cough productive of sputum for at least 3 months.
- Arthritis – self-report of joint problems that interferes with activities of daily living.
- Eyesight problem – self-report of eyesight problems that interferes with activities of daily living.
- Hearing difficulties – self-report of hearing problems that interferes with activities of daily living.
- Skin disorders – self-report of skin problems that interferes with activities of daily living.
- Gastrointestinal disorders – self-report of gastrointestinal disorder that interferes with activities of daily living.
- Dementia – meeting the diagnostic criteria for dementia according to either DSM-IV (Diagnostic and Statistical Manual of Mental Disorders), or the cross-culturally validated 10/66 dementia diagnosis algorithm [23, 24].
- Depression – Mild, moderate or severe depressive episode according to ICD-10 (International Classification of Diseases) criteria.
- Anxiety symptomatology – GMS (Geriatric Mental State Examination) and its associated diagnostic algorithm: AGECAT (Automated Geriatric Examination for Computer Assisted Taxonomy), where a score of 3 or above on the stage I anxiety axis indicated the presence of anxiety [25].

Multimorbidity as an exposure is treated as a count variable (i.e. 0–15 conditions) for estimation of associations with incident care dependence. However, a supplementary analysis is provided where multimorbidity is defined as a binary variable, since multimorbidity is most commonly defined as two or more conditions. For the analysis of the independent effect of physical multimorbidity, mental and cognitive disorders, a physical multimorbidity variable was constructed, as the count of the 12 physical health conditions (i.e. all the above except depression, anxiety and dementia).

Age, gender, education, assets and food insecurity were included as potential sociodemographic confounders. Detailed description of how these data were collected is provided elsewhere [22, 23]. A household asset index was used to assess wealth - a better indicator than income in older people, who may not have a regular source of income [26]. Food insecurity as a proxy for poverty has been used in previous 10/66 studies [27].

Care dependence was assessed using open ended questions. These questions were presented to a key informant, identified by the interviewer as the person who knows the participant best. The key informant is asked a series of open-ended questions about the participant’s care needs, and the interviewer coded the participant as requiring no care, care some of the time or much of the time [6]. The same method was employed to determine dependence at follow-up. To identify probable cases of incident dependence among participants who had died during the follow-up period, a predictive model for incident dependence was developed using variables from the Community Screening Interview for Dementia (CSI-D) informant interview [28], which was available for all participants. For deceased participants this was conducted as part of an informant verbal autopsy interview, and referred to the period before death. The model used age, the total CSI-D informant score and the following items from the CSI-D informant interview: activity, feeding, toileting, dressing and household chores. The predictive model was developed from those who had survived, then applied to those who were deceased at follow-up to predict cases of incident dependence.

### Statistical analysis

**Fig. 1:**
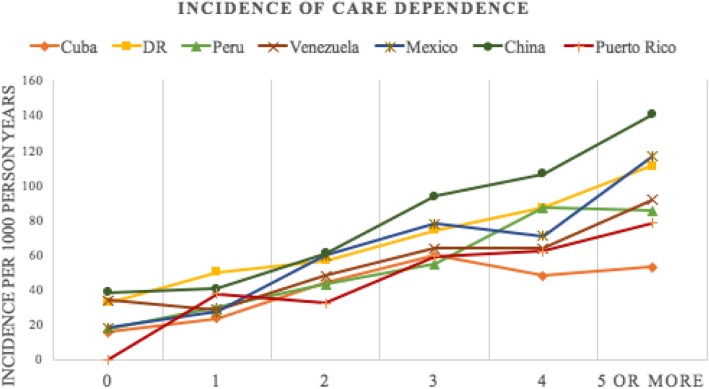
Absolute risk of care dependence by number of chronic conditions

The cohort for analysis of care dependence consisted of participants who were independent of care at baseline. The proportion of dependent free participants at baseline, proportion of dependence-free participants re-interviewed, and the socioeconomic and health outcome profiles of the dependence-free sample and described (Tables 1 and 2). Because the follow-up periods varied slightly between countries, absolute rates of care dependence per 1000 person years are presented (Fig. 1).

**Table 1:** 10/66 incidence wave cohort characteristics

	Cuba	DR	Peru	Venezuela	Mexico	China	Puerto Rico
Baseline cohort							
Baseline cohort	2944	2011	1933	1965	2003	2162	2009
Dependence-free at baseline	2225 (76%)	1770 (88%)	1770 (92%)	1754 (89%)	1807 (90%)	1925 (89%)	1714 (85%)
Missing dependence data	458	4	2	2	0	0	7
Dependence-free cohort at follow-up							
Person years of follow-up	7802	6507	4110	5410	4327	8243	5678
Median follow-up years IQR	4.4 (3.8–5.1)	5.0 (4.4–5.2)	3.2 (2.7–3.7)	4.2 (4.1–4.8)	3.0 (3.0–3.2)	4.9 (4.6–5.3)	4.4 (4.0–4.7)
Alive and reinterviewed %	1660 (75%)	1093 (62%)	1229 (69%)	1154 (66%)	1352 (75%)	1369 (71%)	1154 (67%)
Deceased, informant interview available %	116 (5%)	345 (19%)	78 (4%)	137 (8%)	95 (5%)	384 (20%)	175 (10%)
Refused/not traced %	449 (20%)	332 (19%)	463 (26%)	463 (26%)	360 (20%)	172 (9%)	385 (22%)

*IQR* Interquartile range

**Table 2:** Sociodemographic and health characteristics of cohort at baseline

	Cuba	Dominican Republic	Peru	Venezuela	Mexico	China	Puerto Rico
Characteristics of dependence free cohort who for whom follow-up interviews are available							
Person years of follow-up	8107	5801	3928	4944	4133	7436	5196
Mean age at baseline SD	73.9 (6.4)	74.5 (7.0)	74.2 (7.1)	71.5 (6.1)	73.7 (6.3)	72.7 (5.8)	75.2 (6.5)
Female %	1318 (64%)	940 (65%)	786 (60%)	808 (63%)	922 (64%)	977 (56%)	891 (67%)
Did not complete primary %	389 (22%)	1018 (71%)	253 (19%)	362 (28%)	1000 (69%)	923 (53%)	260 (20%)
0 conditions %	166 (9.3%)	98 (6.8%)	278 (21%)	148 (11%)	186 (13%)	523 (30%)	108 (8.1%)
1 condition %	584 (33%)	364 (25%)	386 (30%)	360 (28%)	401 (28%)	679 (39%)	323 (24%)
2 conditions %	483 (27%)	368 (26%)	290 (22%)	329 (25%)	385 (27%)	332 (19%)	325 (24%)
3 or more conditions %	543 (31%)	608 (42%)	353 (27%)	454 (35%)	475 (33%)	219 (12%)	573 (22%)
Dementia %	73 (4.1%)	124 (8.6%)	58 (4.4%)	34 (2.6%)	71 (4.9%)	38 (2.2%)	50 (3.8%)
Depression %	58 (3.3%)	178 (12.4%)	57 (4.4%)	52 (4.0%)	49 (3.4%)	1 (0.1%)	25 (1.9%)
Anxiety %	66 (3.7%)	107 (7.4%)	85 (6.5%)	89 (6.9%)	65 (4.5%)	3 (0.2%)	55 (4.1%)

*SD* standard deviation

In longitudinal studies of older adult populations, death is often the main reason for losses to follow-up. As the number of participants in the cohort decline over time due to mortality, the risk of care dependence could be overestimated among increasingly fewer participants who lived longer. Competing risk models accounts for this bias when estimating the risk of care dependence. It has been suggested that research in older adults make use of this method [29] though to the authors’ knowledge, it had not been used to assess the association of multimorbidity and dependence.

In this paper where the event of interest is care dependence, the death of a participant competes with the observation of dependence as it “hinders” the event of interest from occurring. Time until the event of interest for participants who became dependent at follow-up was defined as the midpoint between baseline and follow-up assessment. If the participant was deceased and the informant interview revealed care dependence prior to death, time until the competing risk event was defined as the midpoint between baseline and date of death.

The concurrent influence of other factors on the incidence of dependence was examined after adjusting for sociodemographic factors (age, sex, education, wealth and food insecurity), incrementally in four blocks (Models 1–4). To assess the independent effects of physical multimorbidity and mental and cognitive disorders, we first ran a model with physical multimorbidity and care dependence controlling for the sociodemographic factors (Model 5), then adjusted for depression (Model 6), anxiety (Model 7) and dementia (Model 8) individually. Finally, we ran a model with all three mental and cognitive disorders (Model 9). China was excluded from Models 6, 7 and 9, given the low prevalence of depression and anxiety in the China sample (Table 1).

Many participants lived with other participants in the same household (15,027 individual participants from 11,834 households took part in the baseline survey), thus clustering by household was taken into consideration, allowing for intragroup correlation when calculating standard errors.

All analyses were stratified by country, and the estimates for the effect sizes (proportional sub-hazard ratios, with 95% confidence intervals) were then combined using a fixed effect meta-analysis. Inter-country heterogeneity of effect was assessed by Higgins I^2^; 40% or below indicates mild heterogeneity, and 40–60% moderate heterogeneity.

### Ethics

Anonymised 10/66 study data is available through a monitored data sharing repository [30]. Original data collection was granted ethical approval by King’s College London and local institutions.

## Results

In all 15,027 older people took part in the baseline survey, with a target sample size of 2000 participants in each country, and 3000 in Cuba. The response proportion, by country, ranged from 74% in urban China to 95% in the Dominican Republic – further details can be found in a published cohort profile [22]. 12,965 participants were included in the competing risk analysis, as they were independent of care at baseline. 10,341 of the participants’ key informants were interviewed at follow-up and 2624 were lost to follow-up. 498 participants who were deceased at follow up were identified as care dependent before death (Table 1).

Multimorbidity was highly prevalent even in those independent of care at baseline (Table 1). Multimorbidity (two or more conditions) affected between 31% (China) to 68% (Dominican Republic) of the participants (Table 2). Follow-up data showed a linear relationship between count of multimorbidity (0 to 5 or more) and risk of dependence in most countries. In Cuba, this plateaued at 3 or more conditions and in Peru, this plateaued at 4 or more conditions (Fig. 1). Incidence rates became more divergent at higher counts of multimorbidity, with the highest incidence rates in China and the lowest incidence rates in Cuba.

Each increase in count of multimorbidity in the pooled analysis increased the cumulative risk of dependence by 20% (see Table 3), independent of socioeconomic factors. Sociodemographic factors attenuated the effect of multimorbidity slightly (SHR 1.24 in crude model, compared to SHR 1.20 in the fully adjusted model). Most of this is due to the effect of age on the model; the magnitude of this attenuating effect varying somewhat by country (see Table 4 for results stratified by country with multimorbidity as a count variable). Apart from age, no other covariates appeared to reduce the magnitude of the relationship between multimorbidity and care dependence (Table 3). Statistical heterogeneity in the pooled effect size was moderate in the crude model (Model 1, Higgins I^2^ 44%) and negligible after adjusting for age (Model 2, Higgins I^2^ 0%). Similar patterns of association were found when using a dichotomous definition of multimorbidity (see Table 5 and Table 6 for results stratified by country with multimorbidity as a dichotomous variable).

**Table 3:** Meta-analyzed sub-hazard ratios for care dependence with multimorbidity as count variable

	Meta-analysed effect		Higgins I^2^	
	SHR	(95% CI)	%	(95% CI)
Model 1 - multimorbidity				
Multimorbidity	1.24	(1.21–1.27)	44%	(0–77%)
Model 2 - multimorbidity and age				
Multimorbidity	1.19	(1.16–1.23)	0%	(0–71%)
Model 3 - multimorbidity, age and gender				
Multimorbidity	1.19	(1.16–1.23)	0%	(0–71%)
Model 4 - multimorbidity, age, gender, education, asset index and food insecurity				
Multimorbidity	1.20	(1.16–1.23)	1%	(0–71%)
Model 5* - physical multimorbidity				
Multimorbidity	1.11	(1.08–1.15)	38%	(0–74%)
Model 6* - physical multimorbidity and depression				
Multimorbidity	1.08	(1.04–1.12)	37%	(0–75%)
Depression	1.86	(1.52–2.28)	0%	(0–75%)
Model 7* - physical multimorbidity and anxiety				
Multimorbidity	1.08	(1.04–1.12)	0%	(0–75%)
Anxiety	1.57	(1.28–1.92)	35%	(0–74%)
Model 8* - physical multimorbidity and dementia				
Multimorbidity	1.11	(1.07–1.15)	0%	(0–71%)
Dementia	9.29	(7.98–10.8)	50%	(0–79%)
Model 9* - physical multimorbidity and all mental and cognitive disorders				
Multimorbidity	1.08	(1.04–1.12)	0%	(0–75%)
Depression	1.50	(1.20–1.88)	27%	(0–70%)
Anxiety	1.05	(0.83–1.34)	0%	(0–75%)
Dementia	8.72	(7.43–10.2)	58%	(0–83%)

*the model also includes age, gender, education, asset index and food insecurity

*SHR* sub-hazard ratio. *CI* 95% confidence interval

**Table 4:** Univariable and multivariable models of multimorbidity (as count variable) and physical multimorbidity

	Cuba			Dominican Republic			Peru			Venezuela			Mexico			China			Puerto Rico		
	SHR	CI		SHR	CI		SHR	CI		SHR	CI		SHR	CI		SHR	CI		SHR	CI	
Model 1 - multimorbidity																					
Multimorbidity	1.23	1.15	1.32	1.18	1.12	1.25	1.31	1.21	1.42	1.21	1.13	1.28	1.31	1.22	1.41	1.32	1.22	1.42	1.21	1.13	1.29
Model 2 - multimorbidity and age																					
Multimorbidity	1.15	1.06	1.24	1.17	1.11	1.24	1.22	1.12	1.33	1.18	1.11	1.26	1.26	1.17	1.37	1.25	1.14	1.36	1.17	1.09	1.26
Model 3 - multimorbidity, age and gender																					
Multimorbidity	1.15	1.06	1.24	1.17	1.11	1.24	1.20	1.10	1.31	1.19	1.11	1.27	1.26	1.17	1.37	1.25	1.15	1.36	1.16	1.08	1.25
Model 4 - multimorbidity, age, gender, education, asset index and food insecurity																					
Multimorbidity	1.15	1.06	1.25	1.18	1.11	1.25	1.22	1.11	1.33	1.18	1.10	1.27	1.26	1.17	1.36	1.28	1.17	1.40	1.16	1.08	1.25
Model 5 - physical multimorbidity^a^																					
Multimorbidity	1.03	0.94	1.12	1.06	0.98	1.15	1.08	0.96	1.21	1.15	1.07	1.25	1.15	1.05	1.25	1.22	1.11	1.33	1.11	1.02	1.20
Model 6 - physical multimorbidity and depression^a^																					
Multimorbidity	1.01	0.92	1.11	1.01	0.93	1.09	1.05	0.94	1.19	1.13	1.05	1.22	1.14	1.05	1.25	–	–	–	1.10	1.02	1.19
Depression	1.90	1.02	3.53	1.85	1.37	2.49	2.05	1.11	3.82	2.31	1.36	3.91	1.37	0.74	2.56	–	–	–	1.58	0.70	3.56
Model 7 - physical multimorbidity and anxiety^a^																					
Multimorbidity	1.03	0.94	1.13	1.05	0.97	1.13	1.04	0.92	1.17	1.12	1.04	1.22	1.14	1.04	1.24	–	–	–	1.10	1.01	1.19
Anxiety	0.74	0.32	1.67	1.40	0.97	2.01	2.49	1.56	3.96	1.64	1.00	2.69	1.37	0.78	2.40	–	–	–	1.62	0.92	2.85
Model 8 - physical multimorbidity and dementia^a^																					
Multimorbidity	1.05	0.96	1.16	1.07	0.99	1.16	1.07	0.96	1.19	1.13	1.04	1.22	1.11	1.01	1.23	1.20	1.09	1.31	1.12	1.04	1.22
Dementia	11.3	7.96	16.0	10.1	7.54	13.6	9.00	5.92	13.7	3.76	1.99	7.09	11.0	7.76	15.6	6.37	3.56	11.4	8.39	5.22	13.5
Model 9 - physical multimorbidity and all mental and cognitive disorders^a^																					
Multimorbidity	1.05	0.96	1.15	1.03	0.95	1.12	1.04	0.92	1.17	1.10	1.02	1.19	1.11	1.00	1.23	–	–	–	1.12	1.03	1.21
Depression	1.64	0.80	3.35	1.63	1.17	2.26	1.61	0.79	3.30	2.05	1.19	3.53	0.87	0.46	1.64	–	–	–	0.62	0.20	1.89
Anxiety	0.71	0.32	1.60	1.03	0.68	1.57	1.26	0.71	2.23	0.96	0.55	1.66	1.06	0.55	2.04	–	–	–	1.36	0.66	2.80
Dementia	10.5	7.38	14.9	9.58	7.10	12.9	8.07	5.16	12.6	3.35	1.83	6.14	9.92	6.93	14.2	–	–	–	7.61	4.63	12.5

^a^adjusted also for age, sex, education, wealth and food insecurity. *SHR* sub-hazard ratio. *CI* 95% confidence intervals

**Table 5:** Meta-analyzed sub-hazard ratios for care dependence with multimorbidity as binary variable

	Meta-analysed effect		Higgins I^2^	
	SHR	(95% CI)	%	(95% CI)
Model 1 - multimorbidity				
Multimorbidity	2.00	(1.81–2.22)	36%	(0–73%)
Model 2 - multimorbidity and age				
Multimorbidity	1.74	(1.57–1.94)	0%	(0–71%)
Model 3 - multimorbidity, age and gender				
Multimorbidity	1.73	(1.56–1.93)	0%	(0–71%)
Model 4 - multimorbidity, age, gender, education, asset index and food insecurity				
Multimorbidity	1.75	(1.57–1.94)	0%	(0–71%)

*SHR* sub-hazard ratio. *CI* 95% confidence interval

**Table 6:** univariable and multivariable models for multimorbidity (as binary variable)

	Cuba			Dominican Republic			Peru			Venezuela			Mexico			China			Puerto Rico		
	SHR	CI		SHR	CI		SHR	CI		SHR	CI		SHR	CI		SHR	CI		SHR	CI	
Model 1 – multimorbidity^a^																					
Multimorbidity	2.29	1.74	3.02	1.62	1.28	2.04	2.25	1.61	3.15	1.96	1.47	2.63	2.89	2.05	4.08	1.90	1.55	2.33	1.90	1.42	2.53
Model 2 – multimorbidity^a^ and age																					
Multimorbidity	1.83	1.37	2.42	1.59	1.25	2.01	1.79	1.28	2.50	1.76	1.32	2.35	2.43	1.72	3.45	1.64	1.31	2.05	1.64	1.23	2.18
Model 3 – multimorbidity^a^, age and gender																					
Multimorbidity	1.82	1.36	2.43	1.59	1.25	2.02	1.73	1.23	2.41	1.78	1.33	2.38	2.42	1.71	3.44	1.64	1.32	2.05	1.61	1.21	2.14
Model 4 – multimorbidity^a^, age, gender, education, asset index and food insecurity																					
Multimorbidity	1.80	1.35	2.41	1.57	1.23	2.00	1.74	1.24	2.44	1.74	1.30	2.34	2.42	1.70	3.46	1.74	1.38	2.18	1.61	1.21	2.15

^a^binary variable, ≥2 conditions. *SHR* sub-hazard ratio. *CI* 95% confidence interval

**Fig. 2:**
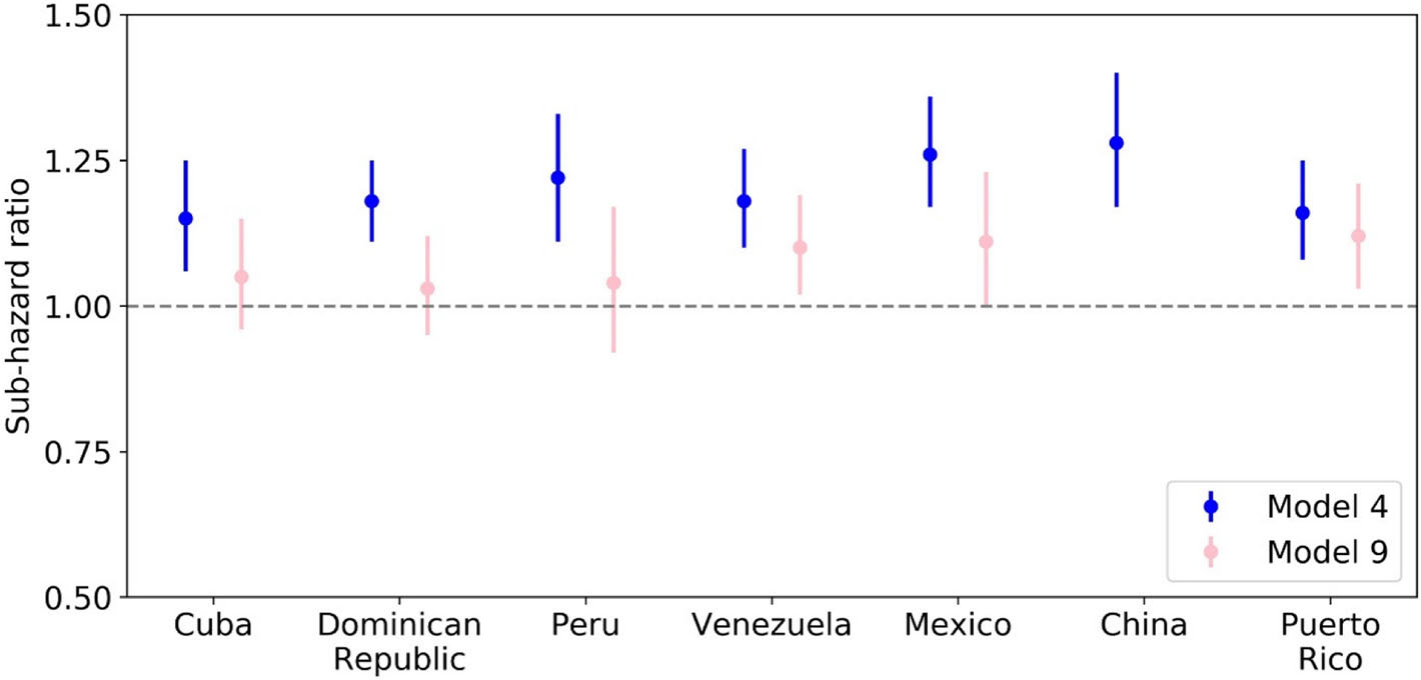
Sub-hazard ratios for dependence per increase in count of total and physical multimorbidity. Model 4 = multimorbidity, age, gender, education, asset index, food insecurity. Model 9 = physical multimorbidity, age, gender, education, asset index, food insecurity, depression, anxiety and dementia. Note that data for Model 9 is not available for China. Y axis shows sub-hazard ratio for total multimorbidity in Model 4 and physical multimorbidity in Model 9

Physical multimorbidity was a significant predictor of care dependence in the pooled effect size but not so in all individual countries. In Cuba, Dominican Republic and Peru the effect fell short of significance (Fig. 2 and Table 4). In all countries and in the pooled analysis, the effect size for physical multimorbidity was smaller than that of multimorbidity with mental health conditions included.

Depression, anxiety and dementia all have significant effects on care dependence independently of physical multimorbidity. This is especially striking for dementia, which increased the risk of dependence nine-fold. Inclusion of depression and anxiety attenuated the relationship between physical multimorbidity and care dependence (see Models 6 and 7 in Table 3), though dementia did not (see Model 8 in Table 3). There was no further attenuation of physical multimorbidity when all three mental and cognitive conditions are added to the model (see Model 9 in Table 3).

## Discussion

Multimorbidity increased the cumulative risk of care dependence, accounting for sociodemographic factors, such that each additional physical/mental health condition increased the risk of dependence by 20% (see Table 3). The impact of age on care dependence varies slightly between countries. However, as shown by the negligible statistical heterogeneity in the age-adjusted models, the association between multimorbidity on care dependence is consistent across all countries despite potential differences in the nature and severity of conditions, or the proportion of undiagnosed conditions which make up multimorbidity in different countries.

China has one of the highest risks of dependence associated with multimorbidity in this study, despite the almost negligible rates of highly burdensome common mental disorders in China (Table 2). Whilst this could be an artefact of the questionnaires used in this study [27], another model for understanding this contradictory finding is that, at the time of the study, China had one of the higher proportions of out-of-pocket payments for government healthcare services amongst the countries in this study [31, 32]. This could potentially deter people from accessing services for milder conditions resulting in only severe and more burdensome conditions being diagnosed. It is worth noting that prevalence of dementia is similar in China as in other countries and the association of dementia with care dependence is much stronger. It is nevertheless interesting that despite some cross-country variance in the make-up of multimorbidity, homogeneity in the outcome is preserved.

Depression and anxiety attenuate the effect of physical multimorbidity on risk of care dependence. The relationship between mental health conditions and physical multimorbidity needs to be interpreted with care as data were collected at the same time. Whilst the attenuation of the effect of physical multimorbidity may reflect confounding, neither can we exclude mediation – a sequence of causation whereby physical multimorbidity brings on a depressive state or anxiety, which causes individuals to become care dependent. Either is theoretically possible and it is not possible to distinguish between the two possibilities without more than the two time points in available in this study.

## Strength and limitations

The study benefited from a longitudinal design, thus limiting information bias and supporting the hypothesis that multimorbidity is a causal factor in the development of care dependence.

The use of a competing risk model allowed for a more representative result that accounted for the high mortality rate (Table 1). However, the method used to identify dependence in deceased participants differed from that used on participants still alive, which could have led to non-random misclassification of dependence. Nevertheless, the algorithm used to identify dependence in deceased participants was based on questions commonly used to assess disability in activities of daily living that are likely to have led to needs for personal care.

There are several issues regarding the multimorbidity variable which could affect the outcome. First, the total multimorbidity variable relied predominantly on self-report of conditions. This will have resulted in misclassification of the exposure variable, that is most likely random, leading to underestimation of the true association between multimorbidity and care dependence. Second, nearly all physical conditions relied on self-report whereas all mental and cognitive disorders used clinical assessment tools. With respect to comparing the independent effect of physical multimorbidity with the effect of individual mental and cognitive disorders, the latter may have been assessed with more precision, leading to a potentially spurious conclusion that the contribution of mental and cognitive disorders is greater than that of physical multimorbidity. Finally, all physical conditions are grouped into one variable; thus, studies that examine individual physical conditions may have different findings.

The study only included people above the age of 65, of whom around 1 in 10 were care dependent at baseline, and hence excluded from the cohort. It is possible that pathway leading from multimorbidity to dependence started earlier in life, in which case the risk conferred by multimorbidity has not been fully captured.

## Contextualization with other research

It is unsurprising given the association of chronic conditions and care dependence [6] that multimorbidity leads to an increased risk of care dependence. We only identified one other longitudinal study that examined the risk of care dependence in relation to multimorbidity. This German study found a hazard ratio of 1.41 for older adults with multimorbidity at five years (where multimorbidity is defined as three or more conditions). As in this paper, dementia conferred a much higher risk for care dependence than the general multimorbidity variable [16]. Other similar studies including one using the same sample as this paper, examining the incidence of care dependence in relation to frailty; this association was attenuated by physical and mental health comorbidities [14]. Another study in Iceland examining both frailty and multimorbidity with the outcome of nursing home admission finding that for frailty alone, the hazard ratio was 1.13, and for those with frailty and multimorbidity, the hazard ratio increase to 2.10 (where multimorbidity was defined as two more conditions) [33]. These studies support the finding in this paper that there is an independent effect of multimorbidity on future care dependence in both LMIC and HIC settings.

There is other evidence that conditions of the mind and brain, especially dementia, contribute a great deal to care dependence [6, 16], and that geriatric conditions, including depression and cognitive impairment are stronger predictors of disability than physical multimorbidity [12]. Worldwide, and across all age groups, mental health disorders are leading causes of disability [34].

Our study explores and supports a pathway by which mental health conditions exert their effect independently of physical multimorbidity. In addition to the independent effect of mental disorders, some of the effect of physical multimorbidity might also be mediated through depression and anxiety as physical multimorbidity was somewhat attenuated after controlling for these two conditions. Whether the relationship is that of mediation or confounding will require further research.

Dementia did not attenuate the effect of physical multimorbidity on care dependence. In contrast, other studies have found that the interplay between multimorbidity and dementia cause a faster progression of cognitive decline that in turn results in care dependency [35]. There are other factors which likely play a key role in the pathway from multimorbidity to care dependence such as widespread polypharmacy amongst people with multimorbidity [36], and the increased risk of morbidity associated with polypharmacy [37, 38]. It could be argued that knowledge on pathways and interactions in multimorbidity should be based on research conducted locally; for example, research using the syndemics framework [39] has revealed synergistic interactions between comorbidities and sociocultural factors idiosyncratic to specific populations [40], highlighting limitations in generalizing interactions and burdens of multimorbidity across different settings.

## Implications for policy and research

Multimorbidity, as measured by a count of chronic physical, mental and cognitive conditions, is a robust and consistent predictor of the onset of care dependence. Like many other studies on multimorbidity in LMICs, this study relies largely on self-report [31, 41]. In LMICs, where there is more underdiagnosis of conditions and less comprehensive medical or health insurance records, reliance on self-report may be the more practical option. Stronger associations may have been demonstrated if diagnoses were confirmed or screened for in this study e.g. blood tests for HbA1c for diabetes or echocardiograms for cardiac disease, but there would be limited potential for translating this into practice. This study has demonstrated that, despite the heterogeneity expected from self-report, the association between multimorbidity and care dependence across different countries is remarkably homogenous.

The relatively simple method employed in this study can help identify at-risk individuals who might benefit from comprehensive geriatric assessment and care, with the aim of optimizing and integrating management, maintaining intrinsic capacity, and reducing functional decline [42]. Whilst it might be argued that in LMIC settings, frailty is a more conveniently assessed risk stratifier, the method of measuring multimorbidity in this study, based mostly on self-report, is of equivalent convenience and practicality to frailty measures. The Integrated Care for Older Persons (WHO-ICOPE) guidelines provide an evidence base and a structure for community-based programs built around these principles, with the explicit aim of improving coverage of age-appropriate services in low and middle income countries [43, 44]. To maintain autonomy and achieve a compression of morbidity across the world, it is imperative that a holistic approach is taken that addresses physical, mental and cognitive health and wellbeing [11, 21, 45].

## Conclusion

Multimorbidity consistently predicts care dependence in China and Latin America with little variation among countries. Age reduces the magnitude of this relationship. Physical multimorbidity imparts a lower risk than multimorbidity with mental health conditions included. Mental health conditions independently increase the risk of care dependence. Depression and anxiety also attenuate the relationship between physical multimorbidity and care dependence. Holistic assessment of physical and mental health conditions can help identify individuals at high risk of care dependence.

## Abbreviations

LMIC: Low and middle income country/countries
HIC: High income country/countries
WHO-ISH: World Health Organization/ International Society of Hypertension
COPD: Chronic obstructive pulmonary disease
DSM-IV: Diagnostic and Statistical Manual of Mental Disorders IV
ICD-10: International Classification of Diseases 10
GMS: Geriatric Mental State Examination
AGECAT: Automated Geriatric Examination for Computer Assisted Taxonomy
CSI-D: Community Screening Interview for Dementia
IQR: Interquartile range
SD: Standard deviation
SHR: Sub-hazard ratio
CI: Confidence interval
WHO-ICOPE: World Health Organization Integrated Care for Older Persons
